# Rethinking schizophrenia in the context of normal neurodevelopment

**DOI:** 10.3389/fncel.2013.00060

**Published:** 2013-05-15

**Authors:** Vibeke S. Catts, Samantha J. Fung, Leonora E. Long, Dipesh Joshi, Ans Vercammen, Katherine M. Allen, Stu G. Fillman, Debora A. Rothmond, Duncan Sinclair, Yash Tiwari, Shan-Yuan Tsai, Thomas W. Weickert, Cynthia Shannon Weickert

**Affiliations:** ^1^Schizophrenia Research Laboratory, Schizophrenia Research InstituteSydney, NSW, Australia; ^2^Neuroscience Research AustraliaSydney, NSW, Australia; ^3^School of Psychiatry, University of New South WalesSydney, NSW, Australia; ^4^School of Medical Sciences, University of New South WalesSydney, NSW, Australia; ^5^School of Psychology, Australian Catholic UniversitySydney, NSW, Australia

**Keywords:** cognition, neurogenesis, myelination, excitatory synapses, neural migration, NMDA receptor, GABA receptor, dopamine receptor

## Abstract

The schizophrenia brain is differentiated from the normal brain by subtle changes, with significant overlap in measures between normal and disease states. For the past 25 years, schizophrenia has increasingly been considered a neurodevelopmental disorder. This frame of reference challenges biological researchers to consider how pathological changes identified in adult brain tissue can be accounted for by aberrant developmental processes occurring during fetal, childhood, or adolescent periods. To place schizophrenia neuropathology in a neurodevelopmental context requires solid, scrutinized evidence of changes occurring during normal development of the human brain, particularly in the cortex; however, too often data on normative developmental change are selectively referenced. This paper focuses on the development of the prefrontal cortex and charts major molecular, cellular, and behavioral events on a similar time line. We first consider the time at which human cognitive abilities such as selective attention, working memory, and inhibitory control mature, emphasizing that attainment of full adult potential is a process requiring decades. We review the timing of neurogenesis, neuronal migration, white matter changes (myelination), and synapse development. We consider how molecular changes in neurotransmitter signaling pathways are altered throughout life and how they may be concomitant with cellular and cognitive changes. We end with a consideration of how the response to drugs of abuse changes with age. We conclude that the concepts around the timing of cortical neuronal migration, interneuron maturation, and synaptic regression in humans may need revision and include greater emphasis on the protracted and dynamic changes occurring in adolescence. Updating our current understanding of post-natal neurodevelopment should aid researchers in interpreting gray matter changes and derailed neurodevelopmental processes that could underlie emergence of psychosis.

## Introduction

A recent perspective titled “Rethinking Schizophrenia” (Insel, 2010) forecast that if researchers and clinicians approached schizophrenia as a neurodevelopmental disorder, with several discernible disease stages, then prevention focusing on the pre-psychotic illness phase would be a real possibility by 2030. The perspective (Insel, 2010) relied on the popular neurodevelopmental theory that the consequences of genetic predisposition and early adverse events, such as mid-gestational insults, would be latent throughout the first two decades of life and become evident as psychosis in early adulthood as first proposed by Weinberger (1987). Weinberger emphasized that the normative maturational changes “unmasked” an earlier insult. While we agree that adolescence is a critical period and that pre- and perinatal development are potential vulnerable time periods when schizophrenia susceptibility genes and environments may contribute to the future onset of schizophrenia, current data indicate human neurodevelopment is not confined to the womb, but is a protracted process that continues in post-natal life well into adolescence and early adulthood. Thus, there is opportunity for perturbation of developmental processes beyond the fetal and perinatal period and, we suggest that developmental disruption well after birth may also contribute to onset of schizophrenia. Our emphasis on the importance of post-natal events is similar to what another schizophrenia neurodevelopmental theorist, Feinberg, originally proposed (Feinberg, 1983). Feinberg suggested that exuberant synaptic regression (“pruning”) in adolescence may underlie schizophrenia, a theory that has dominated our thinking about abnormal neurodevelopment in adolescence in schizophrenia for over 25 years. One of the attractions of Feinberg's hypothesis is that it seemingly fits with the emerging consensus that progressive brain changes in adolescence or early adulthood, thought to be related to tissue loss, are associated with schizophrenia, at least in some individuals (Borgwardt et al., 2009). While we suggest that Feinberg's over-exuberant synaptic pruning theory of schizophrenia is potentially flawed, as it relies on the timing of normative synaptic pruning with inaccurately extrapolated synaptic densities that do not appear consistent with more recent studies, we do submit that charting normal adolescent brain changes is essential if we are to understand the processes underlying schizophrenia pathology. Indeed, a better understanding of changes occurring in the normal adolescent brain may serve to unite protagonists of neurodevelopmental and of neurodegenerative pathologies of schizophrenia. Also, many more molecular studies on human cortical growth and development in the post-natal periods have appeared since the 1980s and the results of these studies, which show diverse and dynamic post-natal changes, deserve to also be incorporated into our working models of the physical substrates of brain maturation in humans and into our theories about abnormal neurodevelopment in schizophrenia.

Here we review current normative neurodevelopmental studies and place them in the context of what is known about schizophrenia neuropathology. We start by considering when emergent behavioral properties of the human prefrontal cortex, like executive function, mature in humans; as prefrontal cortex function is greatly impacted in schizophrenia. Next we consider the timing of cognitive decline in people with schizophrenia, as this can implicate distinct neurobiological events in the pathophysiology. Then we review the temporal development of the “hardware” of the brain; the genesis of new neurons, their journey to the cortex, myelination of axons, and growth of dendritic arbors. Changes in excitatory and inhibitory synapses are reviewed next. We then consider how changes in neurotransmitter receptors and psychopharmacological responses to drugs across development might provide clues to the developmental processes gone astray in schizophrenia. Although there are well-documented differences in the average age of onset of schizophrenia in males (earlier) as compared to females, our review does not cover gender differences in neurodevelopment because our main vantage point is transcriptional and gender differences account for relatively little (130 transcripts = 8%) of the variance of gene transcript levels in a large microarray study of a developmental brain collection and typically occur early in life, not during adolescence (Weickert et al., 2009; Kang et al., 2011).

## Cognitive development

Basic cognitive functions, such as selective attention and response selection are firmly established by early childhood. However, the development of complex executive functions are characterized by a protracted development well through adolescence (Levin et al., 1991; Anderson et al., 2001; Brocki and Bohlin, 2004). Executive functioning capabilities include keeping and manipulating information in short-term memory (also known as working memory), planning, ignoring irrelevant information (involving inhibition and cognitive control), solving problems, and applying existing information to novel situations to derive new solutions (also known as reasoning). Schizophrenia, a disease which typically has its onset during late adolescence or early adulthood, is coincident in time with cognitive maturation of the prefrontal and parietal cortices. This section will: (1) review the timing of the development of executive functions and relationship to measures of neural maturation; (2) place the prefrontal associated cognitive and neural dysfunction accompanying schizophrenia in a developmental perspective; and (3) consider the evidence regarding the timing of cognitive decline in schizophrenia.

### Neurodevelopment of executive functioning

We will consider three aspects of executive processing (working memory, cognitive control, and reasoning), which are separate constructs with differing developmental trajectories and with both distinct and overlapping neural substrates (Brocki and Bohlin, 2004; Huizinga et al., 2006). During the attainment of increased working memory capacity as children get older the superior frontal sulcus (prefrontal cortex) and the intraparietal sulcus (parietal cortex) show a greater degree of task-related activation and have increased white matter connectivity (Klingberg et al., 2002; Kwon et al., 2002; Olesen et al., 2003; Nagy et al., 2004; Edin et al., 2007). When manipulation of information in working memory is required (such as repeating remembered information in reverse order), children perform more poorly and show less activation of the dorsolateral prefrontal cortex (DLPFC) and superior parietal cortices than adolescents and young adults during the same task (Crone et al., 2006). Conversely, other brain regions that are activated during working memory tasks in children, such as the ventromedial prefrontal cortex, show decreased activity in adolescence and early adulthood during working memory tests (Scherf et al., 2006). Thus, in some prefrontal (dorsal) and parietal regions children typically show less activity than adolescents and young adults during executive function/working memory tests; whereas in the ventral prefrontal cortex the pattern is reversed. This suggest that within the frontal lobes, there may be a preference to use more ventral areas to solve working memory problems initially, but this function may be taken over by more dorsal areas of the prefrontal cortex later in life.

**Figure 1:**
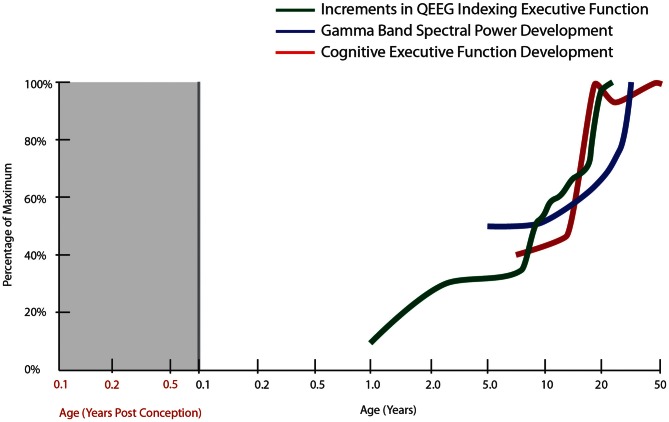
**Normal developmental trajectory of cognitive executive function.** The trajectory of cognitive executive functioning development is derived from developmental tables in the Wisconsin Card Sorting Manual [modified from Heaton et al. (1993)]. The other trajectories correspond to the spectral power in gamma bands during a face recognition task (30–75 Hz, all electrodes; Uhlhaas et al., 2009) as well as cumulative increments in computer quantified changes in EEG frequency spectrum (QEEG) between fronto-temporal cortices, derived from Hudspeth and Pribram (1990).

Another aspect of executive function is cognitive control. Cognitive control relies on a broad group of mental abilities that work together in context to “allow information processing and behavior to vary adaptively from moment to moment depending on current goals, rather than remaining rigid and inflexible” and is thought to rely heavily on the prefrontal cortex (http://carterlab.ucdavis.edu/research/control.php, accessed 7 April 2013). Cognitive control increases as humans mature from childhood to adulthood and this increasing ability correlates with increases in myelination of white matter tracts between the frontal and parietal cortices (Fair et al., 2007; Spear, 2007). The Wisconsin Card Sorting test, a classic test of cognitive flexibility and control, which also relies on fronto-parietal function, shows a developmental pattern of gradual performance improvement interspersed with periods of accelerated improvement throughout childhood to young adulthood (Heaton et al., 1993; Figure 1). Other aspects of cognitive control involve performance monitoring, which is associated with dorsal anterior cingulate activity in adults. Similar to under activation of the dorsal frontal cortical regions in working memory performance, children and adolescents display less dorsal anterior cingulate activity during performance monitoring (Velanova et al., 2008). The protracted development of mature cognition has been linked to relatively late maturation of several association cortices, as compared to other cortical regions such as the sensorimotor and occipital lobes (Giedd et al., 1999; Casey et al., 2000; Gogtay et al., 2004; Blakemore and Choudhury, 2006). We suggest that while a developmental increase in task-related activity of the dorsal prefrontal cortex may be critical to executive function that is typical of adults, this dorsal prefrontal cortex functional maturation co-occurs with functional maturation of other associative regions.

Reasoning (also referred to as a form of intelligence) is thought to rely to a large extent on “executive” processing again residing primarily in the frontal lobes. The two types of reasoning, crystallized and fluid, have somewhat different developmental trajectories. Crystallized intelligence refers to use of previously acquired knowledge to solve problems; whereas fluid intelligence refers to the ability to think logically and solve problems in novel situations independent of previously acquired knowledge. Fluid reasoning shows a rapid increase in ability from mid-childhood until early adulthood, whereas crystallized reasoning ability grows gradually, peaking in mid-life (Cattell, 1987; McArdle et al., 2002). The rostrolateral prefrontal cortex is consistently associated with logical reasoning ability (Prabhakaran et al., 1997; Christoff et al., 2001; Kroger et al., 2002), which also involves activation of other cortices that can vary depending on task demands (Bunge et al., 2005; Wright et al., 2008; Crone et al., 2009). While even toddlers (3-years old) can solve simple reasoning problems (Goswami, 1989), accurate performance on more difficult reasoning problems only occurs in later childhood to adolescence (Sternberg and Rifkin, 1979; Richland et al., 2006). During difficult reasoning problems rostral prefrontal cortical activity is delayed or fails to be sustained in children (Bunge et al., 2009; Crone et al., 2009). Thus, during difficult reasoning problems the timing and maintenance of activity increases as performance increases from early childhood to young adulthood. It is clear from these three examples of cognitive functional change during human development that most executive cognitive processes involve the ability to achieve and maintain activation of focal frontal regions in appropriate contexts. This suggests that molecular and cellular maturation in circuitry controlling the magnitude, extent and co-ordination of pyramidal neuron firing across association areas would be expected to increase during childhood and attain near adult levels during adolescence.

Mature ability to meet executive challenges of the adult are associated with more efficient prefrontal cortical activity (Casey et al., 1997; Bunge et al., 2002; Tamm et al., 2002; Durston et al., 2006). The increasing ability of the brain to transfer information more efficiently across widely distributed neural networks is an important part of adolescent development and likely involves faster and more synchronized axonal firing across long distances. *In vivo* brain imaging findings of macro-level increases in white matter (Snook et al., 2005; Liston et al., 2006; Eluvathingal et al., 2007; Giorgio et al., 2008) are thought to reflect progressive myelination at the micro-level (see Myelination section). However, while increased activity in frontal-parietal regions is a general rule throughout development from child to adulthood, for some tasks such as those requiring response inhibition, the lateral prefrontal cortex may show decreased activity (reflecting increased neural efficiency) as development progresses from children to young adults (Fair et al., 2007).

In general, based on its rich connections with other cortical and subcortical structures, the prefrontal cortex is also ideally suited to the task of coordinating activity within the neural network to facilitate increased neural efficiency and improved executive function. Electroencephalography (EEG) data show age-related changes in neural oscillations and synchrony that support enhanced temporal coordination of distributed cortical processes throughout development (Uhlhaas et al., 2009; Figure 1). Interestingly, this work also suggests a period of destabilization during adolescence, followed by reorganization during young adulthood (18–21 years of age), which is characterized by increases in gamma-band power, theta and beta band synchrony. In fact, EEG has detected several region-specific growth spurts (brief periods of accelerated neural development): the first typically occurring in toddlers, a second in early school age children, a third during puberty and early adolescence, and a final growth spurt in young adulthood (Hudspeth and Pribram, 1990, 1992; Figure 1). Thus, important changes in physiological and structural parameters may occur by gradual changes interspersed by occasional rapid increases, two distinct patterns of change that can also be detected with molecular markers, especially for inhibitory interneurons (see later sections of this review). These physiological changes parallel the patterns found for cognitive development where gradual change can be interspersed with brief periods of accelerated cognitive development (Thatcher, 1991, 1992, 1994). The other major point to consider is that while task-related activity of the prefrontal cortex increases in development, it appears that this activity must be integrated and coordinated with other regions and that both an increase in focal prefrontal activity and synchrony of this region with other association cortices may occur during adolescence. Thus, adolescence is a critical window for the organization and functional adjustment of cortical circuitry rendering this time of life particularly sensitive to disruptive effects. Given that the typical emergence of schizophrenia is during late adolescence or early adulthood, these later developmental changes, which may represent vulnerable periods, become especially relevant for the pathophysiology of schizophrenia, where abnormal patterns of oscillatory brain activity, especially in the gamma range, are observed in patients (Uhlhaas et al., 2008).

### Executive function and prefrontal cortex development in schizophrenia

One of the most debilitating problems for people with schizophrenia are the enduring cognitive deficits (Green, 1996), which are often unresponsive to antipsychotic medication (Heinrichs and Zakzanis, 1998; Goldberg et al., 2007). In terms of cognitive dysfunction, the most consistent findings are within the domains of executive function, working memory, inhibitory control, and reasoning (Weinberger et al., 1986; Goldman-Rakic, 1994; Weickert et al., 2000a; Silver et al., 2003; Ravizza et al., 2010). Almost three decades of functional and structural neuroimaging studies in schizophrenia provide converging evidence of localized abnormal activity and connectivity of the prefrontal cortex (Weinberger et al., 1986; Andreasen et al., 1997; Manoach et al., 1999, 2000; Barch et al., 2001; Meyer-Lindenberg et al., 2001; Perlstein et al., 2001, 2003; Callicott et al., 2003; Tan et al., 2006; Potkin et al., 2009). The concomitant refinement of cognitive executive processes, the physical maturation of neural circuitry underlying executive function, and the onset of schizophrenia in adolescence or early adulthood suggests that a failure in these maturational processes may play a critical role in the pathophysiology of schizophrenia.

One of the key questions for our field is when precisely do cognitive problems begin in people with schizophrenia? This is thought to be important as it may help point to the culprit neurodevelopmental event gone awry in the disease. Population and birth cohort studies show that some premorbid individuals displayed lower cognitive ability during childhood and adolescence. Significant deficits in premorbid IQ can be evident by the age of 16 years in people who subsequently develop schizophrenia (Dickson et al., 2012) and may precede the prodromal period (Woodberry et al., 2008; Khandaker et al., 2011). Overall group deficiencies in cognitive ability may be detectable before 8 years of age in people with schizophrenia (Jones et al., 1994; Cannon et al., 2002a; Seidman et al., 2013), and there is a relationship between the size of the IQ decrement and risk of subsequent schizophrenia (Khandaker et al., 2011; Schulz et al., 2012). However, not all people with schizophrenia start off with lower cognitive ability as a child; there is evidence showing that it is adolescent cognitive development in particular that is subject to perturbation in at least 40% of men and women later diagnosed with schizophrenia (Reichenberg et al., 2005). Additionally, there is a well-established 10 point drop from fairly normal pre-morbid IQ estimates in many adults with schizophrenia (50%), which demonstrates that many people with schizophrenia experience healthy childhood cognitive development, with only about 25% having a low premorbid IQ (Weickert et al., 2000a). The nature of the post-childhood schizophrenia-related cognitive declines affecting prefrontal-related verbal and executive abilities in particular (David et al., 1997; Bhojraj et al., 2010; Maccabe et al., 2013) suggests a deviation in the normal adolescent neurodevelopmental processes that typically support improvements in cognitive ability and efficiency with increasing age. We suggest that there may be at least two different patterns of cognitive decline in people who are destined to develop schizophrenia. One pattern where cognitive decline is found early in life and intellectual impairment is widespread, implying that early developmental processes occurring before 8 years of age may have gone awry (Weickert and Goldberg, 2000). However, the modal pattern of cognitive decline in people with schizophrenia is one of decline that is restricted to the second decade of life and may include more exaggerated loss of prefrontal associated cognitive functions (Weickert and Goldberg, 2000). Therefore, it may be informative to consider the underlying neurobiology in the context of alterations of developmental trajectories in later maturing executive functions in order to adequately address the risk factors for those people with schizophrenia demonstrating an adolescent IQ drop. There is some debate about whether premorbid cognitive changes represent liability- or disease-related processes. Whichever is the case, the disease-specific cognitive dysfunctions manifesting during adolescence (Heinrichs and Zakzanis, 1998) and prefrontal, temporal, and parietal cortical gray matter deficits associated with the prodrome (Wood et al., 2008) and schizophrenia itself (Cannon et al., 2002b) may be separate from or additional to the early childhood cognitive dysfunctions found in the subset of people who go onto develop schizophrenia.

The current literature suggests that brain changes in schizophrenia, if apparent premorbidly, are prominent in prefrontal and associative cortical regions and that they correlate with premorbid executive dysfunction. Structural brain changes present in adolescence or young adults during the early transition to illness provide further support for this view (Pantelis et al., 2003). The findings described above suggest a period of later development (e.g., adolescence) when neural perturbations may result in schizophrenia. Therefore, we suggest that schizophrenia should be considered in the context of alterations in the developmental trajectories of these executive functions and their underlying dynamic changes in neural substrates to adequately address the risk factors for aberrant neurodevelopment of schizophrenia. The remainder of this review will address the timing of normal prefrontal cortical development at the cellular and molecular level and its potential to inform us about the cause of neurobiological changes found in the cortex of people with schizophrenia.

## Early events in the formation of the human cortex

Appropriate genesis and migration of neurons are essential building blocks for a healthy brain. Relatively recent studies challenge dogma by suggesting that for some neuronal subtypes these processes may be ongoing throughout development and into adulthood (Gould et al., 1999; Weickert et al., 2000b; Bernier et al., 2002; Fung et al., 2010, 2011a; Wang et al., 2011). This has implications for adolescent-onset neurodevelopmental disorders, such as schizophrenia, as aberrations in these processes could be contributing to disease. Thus, while we begin with early events, we suggest that some of these early processes are not completely exhausted in post-natal life.

### Neurogenesis and neuronal migration

When the nervous system begins, pluripotent cells from the outer embryonic layer, the ectoderm, produce neuroepithelial cells of the neural plate that invaginates to form the neural tube. The most rostral region of this tube swells to form the telencephalon with the lumen eventually becoming the lateral ventricles of the cerebral hemispheres. The telencephalon forms the cerebral cortex, basal ganglia, and hippocampus (Kandel et al., 2000). One of the most important divisions of the telencephalon relevant to schizophrenia neuropathology is the distinction between the dorsal (pallium) and ventral (subpallium) neurogenic zones as they by and large give rise to the two different neuronal types of the cortex. These zones are not only defined by their position, but by expression of distinct molecules [reviewed by Corbin and Butt (2011)]. Progenitors from the dorsal telencephalon spawn all the cortical excitatory pyramidal neurons that migrate radially to the cortical layers (Rakic, 1972). The ventral neurogenic zone gives rise to most of the cortical inhibitory neurons (Anderson et al., 1997; Tamamaki et al., 1997; Ayala et al., 2007) that migrate tangentially to reach the cortex (see below). Recent studies suggest that some inhibitory (GABAergic) interneurons in the human and non-human primate cortex, particularly many of those expressing calretinin, could also arise from progenitors within the dorsopallial proliferative zone (Letinic et al., 2002; Rakic and Zecevic, 2003; Fertuzinhos et al., 2009; Petanjek et al., 2009a,b; Zecevic et al., 2011), although it is unclear if these “dorsal” progenitors may be ventral in origin, having migrated to the dorsal neurogenic zone at an earlier developmental stage.

Cells in both germinal zones initially undergo proliferative, symmetric division, giving rise to two daughter cells that both retain their apical process and progenitor identity, resulting in expansion of the progenitor population (Kosodo et al., 2004; Kosodo and Huttner, 2009). The greatly expanded neuronal number and surface area of the human cerebral cortex depends on repeated rounds of symmetric cell division followed by later asymmetric division resulting in committed neuronal precursors. Given that there are not gross abnormalities in the size or shape of the human brain (in most cases of schizophrenia) it is likely that these very early embryonic events of division and early neuronal differentiation are intact in most people who later go on to develop schizophrenia. However, neuronal precursors residing in the ventral subventricular zone (vSVZ: Haubensak et al., 2004) retain the ability to divide and generate neurons well into post-natal human life and into adulthood (see below), and more selective disruption of the processes controlling vSVZ neurogenesis could lead to much more subtle changes impacting development of cortical interneurons. Therefore, disruption of the vSVZ neurogenesis process would be consistent with the pathology of mental illnesses involving GABAergic interneurons and with a post-natal age of onset, like what is found in schizophrenia.

**Figure 2:**
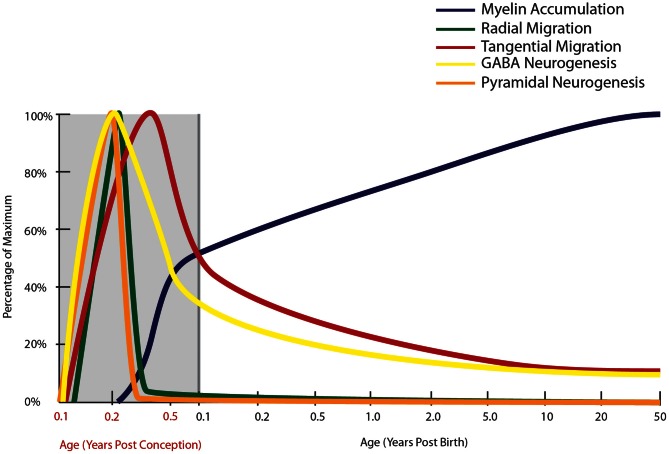
**Normal developmental trajectory of neurogenesis, neuronal migration, and myelination in the human.** Neurogenesis and the subsequent migration of neurons to the cortex begin within a few weeks of gestation in the human (Zecevic et al., 2011). Pyramidal neurogenesis and migration of these cells to the cortex occurs by radial migration and is completed by mid-gestation (Nadarajah and Parnavelas, 2002), while genesis and migration of GABAergic interneurons continues into early post-natal life, with emerging evidence based on the presence of molecular markers of immature neurons suggesting that this process continues into adulthood in primates (Gould et al., 1999, 2001; Bernier et al., 2002; Fung et al., 2011a; Wang et al., 2011). Prefrontal myelination occurs predominantly in early post-natal life, still increasing through adolescence before reaching adult levels (Kang et al., 2011).

Indeed, a deficit in GABAergic inhibition is one of the most robust findings in schizophrenia neuropathology, so the timing of cortical interneuron development warrants further consideration. In the human, GABAergic interneuron birth in the (vSVZ) ganglionic eminence starts as early as 5 gestational weeks (gw: Zecevic et al., 2011) and neurons are found in the nascent human cortex by 5.5–6 gw (Zecevic, 2004). Radial glial cells in the dorsal VZ form around 17 gw. Proliferation of the calretinin positive cell can be detected in the human dorsal SVZ at 20 gw (Zecevic et al., 2011). At 23 gw in the human, proliferation in dorsal VZ proliferation is no longer detectable suggesting that most of the cortical pyramidal neurons are born in embryonic life (Zecevic, 2004; Figure 2). Markers of immature neurons are, however, still abundant in the vSVZ (where cortical interneurons are born) during the first few post-natal years of life (Weickert et al., 2000b; Chong et al., 2008; Wang et al., 2011) and persist into adulthood in humans and monkeys (Fung et al., 2011a; Wang et al., 2011; Figure 2). Neurogenesis has been implicated in schizophrenia pathology by the involvement of disease-associated neurogenesis regulator genes such as DISC1 and neuregulin (Millar et al., 2000; Ghashghaei et al., 2006; Duan et al., 2007; Mao et al., 2009). Current studies using post-mortem material have reported reduced adult neurogenesis in the hippocampus (by PSA-NCAM and Ki67 immunoreactivity: Barbeau et al., 1995; Reif et al., 2006), however, neurogenesis in the vSVZ remains to be studied in schizophrenia.

### Radial migration of neuroblasts from SVZ

After their birth, neurons travel either by radial (dorsal-born pyramidal neurons), or tangential (ventral-born GABAergic interneurons) migration to their final destination in the cortex. Radial migration, via radial glial cells, occurring around 20 gw in humans, is the main mode of migration for pyramidal neurons (Rakic, 1972; Nadarajah et al., 2001; Hatten, 2002; Nadarajah and Parnavelas, 2002; Nadarajah, 2003; Figure 2). Once radial migration of pyramidal neurons ceases, cortical maturation in the form of proper alignment of neuronal cells, synapse formation, and neurite outgrowth lasts from the sixteenth fetal week until the post-natal period (Sidman and Rakic, 1973; Lequin and Barkovich, 1999). One molecular regulator of radial migration and cortical layering, reelin, is an extracellular matrix protein produced by Cajal-Retzius cells in layer I of the cerebral cortex (Marin-Padilla, 1990; Del Rio et al., 1997). In mice lacking normal reelin function (reeler mice), inverted neocortical layers and abnormally dispersed cells are observed due to lack of neuronal ability to respond to migration signals in reeler mutants (Caviness, 1976; Goffinet, 1984; D'Arcangelo et al., 1995; Chai et al., 2009). Reelin is down-regulated in post-mortem brains from people with schizophrenia (Grayson et al., 2005), with significant (approximately 50%) reelin reductions in hippocampus, caudate nuclei, and cerebellum (Impagnatiello et al., 1998; Costa et al., 2002). However, the fact that cortical layering appears to be fairly intact in most cortical areas in schizophrenia, is not consistent with a major loss of function of the reelin gene early in development. Anomalous cortical development due to distorted distribution of neurons, especially in layer II of the entorhinal cortex from patients with schizophrenia has been reported (Jakob and Beckmann, 1986; Arnold et al., 1991, 1997; Falkai et al., 2000) at least in some cases, but not all (Jakob and Beckmann, 1986; Krimer et al., 1997; Bernstein et al., 1998), which could be consistent with more subtle alterations in reelin-mediated migration events than what is found in the reeler mouse.

### Tangential migration of interneurons

In recent decades, there has been an increased understanding of the origins and migration of cortical interneurons (Anderson et al., 1997; Nadarajah and Parnavelas, 2002; Zecevic et al., 2011). During development of the mammalian telencephalon, interneurons migrate tangentially from the ventral ganglionic eminences (vSVZ) to the developing cortex (Corbin et al., 2001; Marin and Rubenstein, 2003; Takemura, 2005). Classically, the migration of cortical interneurons was thought to be completed during fetal life (Sidman and Rakic, 1973; Korr and Schmitz, 1999; Zecevic et al., 2005) and thus the number of interneurons was believed to remain stable throughout life (Spalding et al., 2005; Bhardwaj et al., 2006). While migration of interneurons occurs in the human embryo (Sidman and Rakic, 1973; O'Rahilly and Muller, 1994; Meyer, 2001; Bayatti et al., 2008) and may peak in the midgestational period in humans (Meyer, 2001), recent evidence suggests that it may also continue years after birth. Since we and others find a high density of nascent neurons in their birthplace, the SVZ, and a high neuronal density just below the cortex in human infants (Chong et al., 2008; Fung et al., 2011a; Wang et al., 2011), this suggest that addition of new cortical interneurons may contribute to the large growth of the human brain evidenced by a quadrupling of brain weight (Dekaban and Sadowsky, 1978; Beltaifa et al., 2005) and a large increase in cortical gray matter volume from birth to about 5 years of age (Iwasaki et al., 1997; Shankle et al., 1998a,b; Durston et al., 2001; Lenroot et al., 2007). So, while regressive events have received the bulk of the focus in considering cortical development in the context of schizophrenia there is in fact much larger cortical growth events, which are occurring within the first few years after birth.

**Figure 3:**
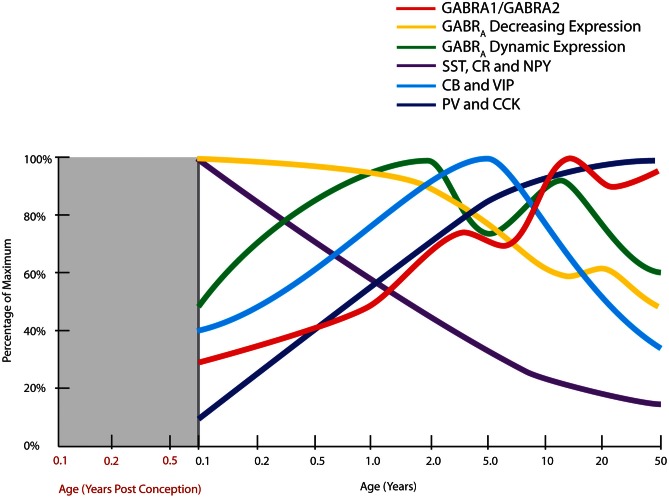
**Normal developmental trajectories of expression of inhibitory system components in the human prefrontal cortex.** Normal development of GABA receptor components of the inhibitory system is dynamic until adolescence where the trajectories reach a steady or declining state. GABA_A_ (GABR_A_) subunits display two distinct patterns, one of decreasing expression following birth (α2, α5, β1, γ1, γ3 subunits) and a M pattern (dynamic expression) with peaks at toddler and teenage time periods (α1, α4, β2, β3, γ2 subunits; Fillman et al., 2010). The peak in the ratio of GABA α1 to α2 subunits (GABRA1/GABRA2) coincides with increased gamma band power in the prefrontal cortex (refer Figure 1). Expression of inhibitory neuron markers display either decline over post-natal life (SST, somatostatin; CR, calretinin; NPY, neuropeptide Y), initial up-regulation and then decline or plateau around school age (CB, calbindin; VIP, vasoactive intestinal peptide), or increased expression over post-natal life (PV, parvalbumin; CCK, cholecystokinin; Fung et al., 2010).

Measurement of biochemical markers representing different interneuron subtypes suggests that parvalbumin and cholecystokinin mRNAs are increased over post-natal development in humans, most strikingly in the first 5 post-natal years (Figure 3). Parvalbumin immunoreactivity first appears in the DLPFC around 3–6 months of age and mRNA is increased 20-fold from neonatal to adult years, indicating that arrival and/or robust maturation of these interneurons occurs in post-natal primate life (Reynolds and Beasley, 2001; Erickson and Lewis, 2002; Cruz et al., 2003; Grateron et al., 2003; Fung et al., 2010). The cell density or mRNA expression of other interneuron markers, such as calbindin and vasoactive intestinal peptide increase similarly in the early post-natal years reaching a peak in children (~4–7 in human) prior to declining to adult levels in adolescence (Yan et al., 1995; Delalle et al., 1997; Fung et al., 2010; Figure 3). Post-natal expression of neuropeptide Y, somatostatin, and calretinin declines with age, indicating that these markers may contribute more to early brain development and become down-regulated with normal brain maturation (Figure 3). For example, density of dendrite-targeting somatostatin immunoreactive cells increases in the non-human primate cortex from E120 to E140, before declining through post-natal life and adulthood (Yamashita et al., 1989; Hayashi et al., 1990), with expression in human being dramatically reduced over the first 10 years of life (Fung et al., 2010). Taken together, these results suggest that recruitment, differentiation, growth, and refinement of cortical inhibitory interneurons is a major developmental event, if not the major developmental event, occurring as cognitive abilities like working memory and language are initially attained.

In addition to the developmental profiles of interneuron markers, high levels of neuronal migration markers such as DCX and poly-sialyated neuronal cell adhesion molecule (PSA-NCAM) in infants (Cox et al., 2009; Fung et al., 2011a; Kang et al., 2011; Xu et al., 2011) provide compelling evidence that cortical interneuron migration continues post-natally, particularly in the first few years of life (Figure 2). In the adult primate brain, new neurons are produced by two neurogenic regions, the sub-granular zone of the hippocampus (Eriksson et al., 1998) and the vSVZ (Curtis et al., 2003), producing granular cells in the dentate gyrus and interneurons thought to be destined for the olfactory bulb, respectively (Kornack and Rakic, 1999, 2001; Gould, 2007). In the rhesus macaque, we have found ~50,000–80,000 dividing cells/12 h along the SVZ of juvenile and adult macaques, using both BrdU and Ki67 positive cell markers of cell proliferation (unpublished; Shalaev et al., 2002). Other studies have demonstrated BrdU and NeuN/GAD_67_ co-labeled cells in the principal sulcus of adult macaques (Gould et al., 1999; Koketsu et al., 2003; Runyan et al., 2006; Ashrafi et al., 2007) suggesting that some of these dividing cells may contribute to an adult generated population of interneurons in the cortex. The abundance of DCX positive cells around the ventricle, and the presence of PSA-NCAM positive neurons in the white matter of the principal sulcus in non-human primate brain, further support post-natal generation of neurons from the vSVZ (Fung et al., 2011a). Additionally, the presence of GAD_65/67_+, somatostatin+ and neuropeptide Y+ neurons with morphology reminiscent of tangentially migrating cells (elongated cell body and leading and/or trailing processes parallel to the pial surface) in the post-natal white matter of primates may indicate the presence of post-natally migrating interneurons (Fung et al., 2011a; Yang et al., 2011; Joshi et al., 2012). A recent study by Alvarez-Buylla and colleagues (Sanai et al., 2011) provides further evidence of a major migratory pathway (DCX+, PSA-NCAM+ cells) in post-natal human brains originating from the SVZ and targeting prefrontal cortex. In primates, there are several reports of immature neurons in the amygdala, and in piriform, inferior temporal and prefrontal cortices in post-natal life (Gould et al., 1999, 2001; Bernier et al., 2002; Runyan et al., 2006; Ashrafi et al., 2007). The recent and emerging post-natal primate studies provide new insight into our still incomplete knowledge of the neurodevelopmental trajectory for tangential neuronal migration and we suggest that cortical interneurogenesis is protracted in humans and may continue at a low level throughout human life and we have incorporated this possibility on our graph (Figure 2).

Interestingly, most biochemical markers of interneurons are reduced in schizophrenia, supportive of an interneuron deficit in the disease. Specifically, there is strong evidence for reduced expression of the calcium binding protein parvalbumin (Beasley and Reynolds, 1997; Reynolds and Beasley, 2001; Reynolds et al., 2002; Hashimoto et al., 2008a,b; Morris et al., 2008; Sakai et al., 2008; Bitanihirwe et al., 2009; Mellios et al., 2009; Fung et al., 2010) and the neuromodulatory peptide, somatostatin (Hashimoto et al., 2008a,b; Morris et al., 2008; Fung et al., 2010). Given the developmental studies highlighting the protracted maturation of interneurons within the primate cortex over a decade or more (see Figure 3) the inhibitory neurons are implicated as a key substrate where brain development may be derailed in schizophrenia, with particular emphasis that a balance of cortical interneuron markers is normally achieved during adolescence (with stabilization of some markers, but down-regulation of others) and overlapping with timing of schizophrenia onset. This requires a new understanding of adolescence as a time of dynamic developmental change, not just as a time of regressive events, but one where the inhibitory system is still being rearranged and positioned for adult life.

There is new evidence that post-natal cortical interneuron migration is of relevance to the pathophysiology of schizophrenia, but this requires a re-interpretation of existing data. Ample evidence for increased interstitial white matter neuron (IWMN) densities in schizophrenia exists (Akbarian et al., 1993; Anderson et al., 1996; Eastwood and Harrison, 2003, 2005a; Kirkpatrick et al., 2003; Yang et al., 2011; Joshi et al., 2012). These observations were initially interpreted as increased remnants of early-generated cortical subplate neurons (a transient population of white matter neurons) derived from the dorsal pallium that failed to undergo programmed cell death (Kostovic and Rakic, 1980; Chun and Shatz, 1989). However, the observations of increased subcortical neurons found in schizophrenia can also be interpreted as being due to abnormal tangential migration of cortical inhibitory interneurons from the vSVZ (Yang et al., 2011; Joshi et al., 2012; Volk et al., 2012). The increase in IWMN density may be indicative of arrested migration of cortical interneurons during their journey to the cortex earlier in life or increased genesis and migration of new interneurons toward the cortex in response to possible cortical trauma, such as increased inflammation (Fillman et al., 2013). In either case, the recognition that cortical interneurons are generated from a unique birth place (vSVZ), require particular neurodevelopmental signals, can take years to establish themselves, and may have the ability to continually turn over, even at low levels, suggests that a greater understanding of the timing, the control and extent of interneurogenesis and migration may provide important clues as to the developmental origins of interneuron pathology in schizophrenia.

## Myelination

Another developmental event that extends well into post-natal life is myelination (Figure 2). Brain axonal tracts are myelinated progressively across the lifespan in a region- and function-specific manner. Early human post-mortem work first described regional differences in the degree of myelination across the lifespan and highlighted the protracted development of a number of white matter tracts. Comparison of qualitative myelin staining in 200 cases from mid-gestation to 1-year old, from 1 to 30-years old, and numerous older cases yielded findings of earlier myelination of the hippocampus, primary motor, and primary sensory areas, and later myelination of frontal cortical white matter, extending into the 4th decade of life (Yakovlev and Lecours, 1967). A follow-up study (*n* = 12 brains) corroborated these findings in the primary motor and sensory cortices, but failed to confirm protracted myelination of the prefrontal cortex (Benes, 1989). Both studies were limited by critical age gaps in the cohort or very small sample sizes at certain stages. A comprehensive larger study graded myelination in 62 white matter sites within 162 post-mortem brains from newborn to 3-years of age (Brody et al., 1987; Kinney et al., 1988). This study confirmed that the major white matter tracts, subcortical regions, primary cortical areas, and hippocampus become myelinated sooner than association cortical areas, but estimated that even in association areas the majority of infants achieve substantial myelination by the age of 2. A more recent study investigated a marker for myelination, myelin basic protein (MBP), in the parietal cortex and found MBP expression commenced at around 3.5 months of age, and reached adult-like levels from 13 months of age (Haynes et al., 2005). Investigation of hippocampal myelination has also suggested the importance of early myelin development, with appearance of oligodendrocytes from gw 20 and of myelinated fibers from post-natal week 2 (Abraham et al., 2010). By 11 years of age, myelinated fiber density in the hippocampus was well developed but had still not reached the highest adult levels (Abraham et al., 2010). These findings suggest that most myelination and axonal growth occur within the first 2 years of early life, but may be ongoing in many telencephalic areas including the frontal cortex and hippocampus. Post-mortem microarray data from our laboratory (Harris et al., 2009; Weickert et al., 2009), have demonstrated dramatic increases in mRNA transcripts of myelin proteins in the prefrontal cortex across human post-natal life. We observed increasing mRNA expression of MBP, myelin-associated oligodendrocyte basic protein (MOBP) and proteolipid protein 1 (PLP1) during the first decade of life (all *p* < 0.001). From birth to 2 years of age, there was an approximately 100 times increase in MBP expression, a 20 times increase in MOBP expression and a 10 times increase in PLP1 expression, followed by a much more gradual increase of all three transcripts from early childhood into their peak in adolescence. These findings are supported by other recent microarray data, in which expression of genes associated with myelination reached approximately 50% of adult levels by birth and 95% of adult levels by 2–3 years of age in the neocortex (Kang et al., 2011). These post-mortem gene expression findings suggest that the most active myelination of the human prefrontal cortex occurs early in life, predominantly in the first 2 years of life, but continues throughout childhood and adolescence before full myelin maturation levels are reached in adulthood.

The molecular findings of developmental myelination trajectories in normal humans have been confirmed with brain imaging studies of growth of white matter tracts across human post-natal development. Throughout the brain, white matter, especially myelin, develops rapidly in the first 12 months of life (with most dramatic changes from 0 to 3 months) followed by slower change from 1 to 2 years and thereafter (Schneider et al., 2004; Dubois et al., 2006; Hermoye et al., 2006; Provenzale et al., 2007; Gao et al., 2009). After the first year of life, and throughout childhood and adolescence, changes in white matter measures (fractional anisotropy) are more subtle and are predominantly due to altered perpendicular diffusivity, reflecting increasing myelination (Giorgio et al., 2008; Lebel et al., 2008; Gao et al., 2009). However, global age-related white matter changes can still be observed in adolescents (Barnea-Goraly et al., 2005; Giorgio et al., 2008, 2010; Bava et al., 2010), while region-specific changes can continue in young adulthood (Giorgio et al., 2008). White matter maturation occurs in a region specific manner, with late maturation associated with the prefrontal cortex throughout the first 2 decades of life reaching full maturation at 18 years (Barnea-Goraly et al., 2005) and fronto-temporal tracts approaching full maturation after 25 years (Lebel et al., 2008).

Overall, these studies provide strong evidence that myelination throughout the brain takes place most rapidly early in life. However, in numerous regions, including prefrontal regions, the process continues throughout the second decade of life with full maturation only reached in late adolescence or young adulthood. In brain regions implicated in the pathogenesis of schizophrenia, such as the prefrontal cortex, full white matter maturation is mostly achieved prior to the period of life when schizophrenia emerges. However, improvements in working memory (which employs the prefrontal cortex) are associated with increased white matter maturation during adolescence (Nagy et al., 2004; Bava et al., 2010), while poorer performance is associated with less white matter maturation (Bava et al., 2010). Deficits of working memory are also consistently seen in schizophrenia (Goldman-Rakic, 1994; Meyer-Lindenberg et al., 2005), and structural abnormalities in white matter underlying the DLPFC have also been reported (Kyriakopoulos et al., 2008). Microarray studies of post-mortem brains from patients with schizophrenia have implicated myelination deficits in the pathology of schizophrenia (Hakak et al., 2001; Matthews et al., 2012). These observations have been validated by targeted investigations of cortex, which have revealed a decrease in mRNA (Matthews et al., 2012) and protein levels (Honer et al., 1999; Parlapani et al., 2009) of MBP in schizophrenia, though not consistently (Beasley et al., 2009). Thus, it seems likely that abnormal maturation of white matter could contribute to cognitive disturbances found in schizophrenia and that a better understanding of the molecular control of white matter maturation and how it could be disrupted in schizophrenia may reveal novel clues to neurodevelopmental origins of schizophrenia.

## Excitatory synapses

Probably the most popular theory for neurodevelopmental origins of schizophrenia is the theory that dendritic spines are over-pruned in adolescence. The formation and elimination of dendritic spines, subcellular structures specialized for receipt and integration of excitatory input to pyramidal neurons is important as spine numbers are thought to correlate with cognitive and learning ability (Elston, 2000).

### Dendritic arborization and spine densities

An early study (Huttenlocher, 1979) quantified synaptic density in the cerebral cortex across the human lifespan and suggested that synaptic regression occurred after the first two post-natal years; however, this study (Huttenlocher, 1979) had only one brain available in the age group between 7 and 25 years of age and one between 25 and 50 years of age, requiring a great deal of extrapolation from few data points. On the basis of the available data, it was concluded that synaptic regression occurs anytime between 2 and 16 years of age in human frontal cortex. It was a few years later that Feinberg interpreted these data as supporting the notion that a physical change, namely “synaptic pruning” could be occurring in the human brain during adolescence, and that emergence of schizophrenia symptomatology may be due to an overexuberance of synaptic elimination (Feinberg, 1983; Keshavan et al., 1994; Selemon and Goldman-Rakic, 1999). While this speculation has had enormous influence on neurodevelopmental models of schizophrenia and is found in most reviews on this topic, few investigators seem to carefully scrutinize the evidence upon which this theory is based, often interpreting a “dotted” line plotting supposed time of synaptic reduction as adolescent pruning, when in fact it is based on only 3 individuals and shows a putative reduction in synaptic density starting several years before adolescence. Further, as synapse densities change with brain volume changes and possible gain or loss of whole neurons, glia and blood vessels, it is not clear if changes in synapse densities across development are attributable to a net loss of number of synapses per neuron (pruning) or changes in these other factors (Huttenlocher, 1990). A subsequent report by Huttenlocher and Dabholkar (1997) with four adolescent cases could suggest in fact that there may indeed be no change or a small net increase in synapse density in the time period between infancy and adolescence in the human prefrontal cortex.

**Figure 4:**
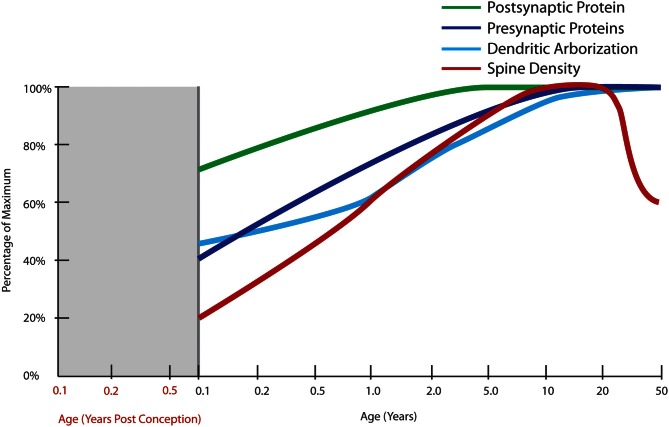
**Normal developmental trajectories of excitatory system components in the human prefrontal cortex.** Normal development of the excitatory system involves increasing expression of presynaptic SNARE complex proteins (SNAP-25, syntaxin 1A, VAMP1) into early adulthood (Webster et al., 2011) and a similar increase in protein expressions of the post-synaptic markers, PSD95 and spinophilin, though starting at a higher baseline at birth (Kang et al., 2011; Webster et al., 2011). Dendritic arborization indexed by average length of the total dendritic tree of layer III pyramidal neurons does not reach its maximum until adulthood with a slight decline after the age of 30 years (Petanjek et al., 2008). Quantification of Golgi-impregnated tissue suggests an increase in spine density from birth until early school age, followed by a period of gradually decreasing density starting in early adulthood which lasts until middle age (Petanjek et al., 2011).

A more recent Golgi study of dendritic development in human cortex by Petanjek and colleagues (2008) spans a broad age range (newborn to age 91 years, *n* = 25) and includes 13 cases between the ages of 1 week and 10 years and studied the development of the layer III basilar dendritic tree from birth to the beginning of adolescence. There was an effect of age on all dendritic variables measured, except for the number of basal dendrites, which remained constant. The dendritic segment count reached adult levels by the age of 12 months. A 3-fold increase in the length of the dendritic tree of layer III pyramidal cells by 2.5 months of age with an additional 50% increase in the length of terminal dendritic segments between 16 and 30 months of age was observed. A plateau in intermediate dendritic segment length was observed during late adolescence, a period which was represented by three cases in this study (Figure 4). These data suggest that the major developmental event during early childhood is exuberant early growth of structures that are specialized to receive synapses and is not consistent with large-scale “pruning” of synapses or synaptic reductions that were believed to occur during post-natal life based on synaptic density counts (Huttenlocher, 1979).

In another study, basilar dendritic complexity appeared greater in younger individuals (14–40 years of age) than in older individuals (40–106 years of age; Jacobs et al., 1997). This was reflected in a statistically significant reduction of 9–12% in total dendritic length and a slight (4%), but statistically significant increase in the number of dendritic branches per cell in the older adult age group compared with the younger age group. The most substantial change observed was in dendritic spine measures, with a gradual 50% decrease in the number and density of spines from the mid-twenties to mid-adulthood (40 years: Jacobs et al., 1997). This suggests that the interpretation of earlier findings of changes in synaptic density reported by Huttenlocher (1979) could have been influenced by the many individuals in the 60–80 year range. However, Jacobs et al. (1997) found the number and density of spines between the ages of 40 to 106 years appeared stable in this study and the variability due to aging was relatively constricted to early adulthood. A subsequent study details changes in spine density in the DLPFC across the lifespan (Petanjek et al., 2011) using a post-mortem brain collection overlapping with that used in Petanjek's study described above. A peak in layer III pyramidal neuron spine densities in early life with higher level in school age as compared to the first year of life and an apparent stability from childhood through adolescence (5–20 years of age), and a change that begins after the second decade of life where spine density appears to gradually decline over the next 30 years (to age 50, Figure 4) and where this loss extends into aging (not graphed).

The studies reviewed above (Jacobs et al., 1997; Petanjek et al., 2008, 2011) had substantial inter-individual variability attributable to differences in the populations studied, and relatively small collection sizes in the context of broad age-ranges of the samples used. Taken together, these studies suggest that the timing of synaptic density change likely involves very early post-natal increases and that the timing and extent of synaptic density decreases and reductions in dendritic spines may be gradual and protracted in humans. The studies available suggest that synaptic pruning may not actually be prominent in human adolescence but may have its onset in toddlerhood until school age and/or begin after adolescence and extend into the 5th decade of life. Thus, the reviewed data suggest that the onset of synaptic regression is unlikely to be the trigger for the onset of schizophrenia. Another way to gather support for the timing of synaptic regression would be to examine quantitative measures of molecular makers of synapses during normal human cortical development as we and others have done and elaborate on next.

### Molecular markers of excitatory synapses

**Figure 5:**
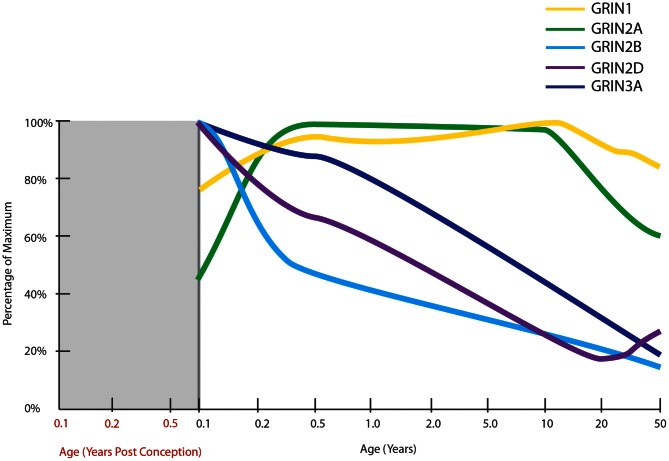
**Normal developmental trajectories of NMDA receptor subunits in the human prefrontal cortex.** Normal development of NMDA receptor subunits (Choi et al., 2009; Colantuoni et al., 2011; Kang et al., 2011). mRNA of the obligatory NMDA receptor subunit, GRIN1 (NR1), is expressed at fairly steady levels post-natally, at least until middle age. GRIN2A expression increases in infancy to attain fairly steady levels throughout childhood and then decreases through adolescence and into adulthood. GRIN2B, GRIN2D, and GRIN3A all have their highest expressions at birth with decreasing trajectories thereafter.

While development of the glutamatergic synapses begins prenatally with the majority of synapses formed during the neonatal period (Feldmeyer and Radnikow, 2009) other work suggests the levels of one protein often used as marker of synapse density, synaptophysin, peaks much later in life during school age years (6–10 years of age) while levels of a protein thought to be marker of dendritic spines, PSD-95, peaks even later, in early adolescence and then marginally declines (Glantz et al., 2007). Consistent with this, Salimi et al. (2008) found a similar pattern of a late peak in the protein expression of complexin 2, a marker of excitatory synapses. Importantly, the ratio of excitatory to inhibitory synapse markers (complexin 2/complexin 1) decreased in adolescence (Salimi et al., 2008), again pointing to the maturation of the inhibitory system as an important adolescent event. Our recent data from multiple molecular markers suggest that developmental increases in synaptic proteins are not followed by a significant decline either in childhood or in adolescence as would be expected with major synaptic regression (Webster et al., 2011). We have found that a variety of synaptic mRNAs and proteins increase steadily after birth during childhood years (Webster et al., 2011). This pattern of increasing expression was found for many presynaptic proteins and was particularly true of the SNARE complex proteins (syntaxin 1A, SNAP-25, and VAMP1) where syntaxin-1A levels peaked in young adulthood or even in adult age (Fung et al., 2011b; Webster et al., 2011; Figure 5). In terms of molecules localized to the postsynaptic elements, PSD-95 and spinophillin both had highest mRNA levels during infancy with protein levels increasing until school age and with no evidence of an adolescent decline in protein (Webster et al., 2011; Figure 5). Overall there is a major developmental increase in synaptic components, especially at the protein level, from neonates through school age and adolescence that remains relatively stable through adolescence and into adulthood. These observations are contradictory to the model proposed by earlier studies that suggested the major developmental event in adolescence is regression or loss of synaptic elements (Huttenlocher, 1979; Feinberg, 1983; Glantz et al., 2007). The work reviewed here provides evidence that the theory stating that exuberant excitatory synapse loss could be the basis for the aberrant adolescent brain development in schizophrenia may need to be reconsidered. This position gains additional support from the lack of consistent molecular changes in presynaptic mRNAs and proteins in schizophrenia brains (see below).

Studies of markers of excitatory synapses in neocortex of patients with schizophrenia primarily find no change (Eastwood and Harrison, 2001, 2005b; Sawada et al., 2002; Halim et al., 2003; Fung et al., 2011b) or slightly decreased levels (Eastwood and Harrison, 2005b). Similarly, measurement of excitatory spine markers has led to divergent results (Law et al., 2004; Weickert et al., 2004; Toro and Deakin, 2005; Kristiansen et al., 2006; Catts and Weickert, 2012), mainly suggesting there is no change or only slight decreased expression of spinophilin and PSD-95 in cortex of patients with schizophrenia. A molecular marker of dendrites, microtubule-associated protein 2, is also typically unaltered in level, but sometimes decreased in the cortex (Jones et al., 2002; Mukaetova-Ladinska et al., 2002; Somenarain and Jones, 2010). Even when slight decreases in molecular markers of excitatory synapses are found, they are typically identified in only a subset of cases (Faludi and Mirnics, 2011), not found in all anatomical regions (Webster et al., 2001; Law et al., 2004; Toro and Deakin, 2005), restricted to certain layers (layer III basilar dendrites only: Glantz and Lewis, 2000; Kolluri et al., 2005) and not consistent across cohorts (Halim et al., 2003; Eastwood and Harrison, 2005b; Castillo et al., 2010; Gray et al., 2010; Fung et al., 2011b). Taken together, schizophrenia, in general, does not appear to be characterized by a pervasive and consistent loss of excitatory synapse markers.

This raises the question of what physical elements could be responsible for cortical gray matter volume reductions found in schizophrenia? A recent and quite comprehensive consideration of the cellular components of cortical gray matter has yielded the following estimates for percentage of space occupied by each: neurons = ~64%, glial = ~12.5%, synapses = ~6%, capillaries = ~0.5%, and extracellular spaces = ~17% (Bennett, 2011). Based on available histological evidence from studies on people with schizophrenia, Bennett suggests that a deficit in spines (Glantz and Lewis, 2000; Kolluri et al., 2005) alone would be insufficient to explain the gray matter loss found in imaging studies which is typically on the order of a 8–10% reduction. The fact that the loss of spines is quite anatomically restricted, i.e., occurs in deep layer III, but not in superficial layer III, and is not found in layers V or layers VI in frontal cortex also supports that spine loss is likely only a partial explanation for cortical thinning (Glantz and Lewis, 2000; Kolluri et al., 2005). Bennett concludes that other elements must be involved and he suggests that loss of dendrites is the likely suspect (Bennett, 2011). However, the 30% loss of dendrites used to form this argument appears to only apply to basilar dendrites as apical dendrites and segment length are actually non-significantly increased in the cortex of people with schizophrenia (Kalus et al., 2000). Considering that basilar dendrites occupy an estimated 60% of the total dendritic length (Soloway et al., 2002), this reported loss of dendrites still may be only a partial explanation for cortical volume loss. What the anatomical substrate of the consistent reductions in gray matter volume found in patients with schizophrenia actually is, if it is not synaptic loss, remains an unanswered question. Clearly more quantitative anatomical studies of the brains of patients with schizophrenia are needed to determine the answer. We suggest that it is premature to consider all gray matter reduction in schizophrenia to be due solely to loss of synapses or to over-exuberant synaptic pruning.

## Receptor systems

Schizophrenia pathophysiology likely involves disruptions to three neurotransmitter systems, namely; excitatory glutamatergic, inhibitory GABAergic, and modulating dopaminergic, input [reviewed in Seshadri et al. (2013)], the developmental trajectories of some of the main receptors for each are reviewed below.

### NMDA receptors

The majority of excitatory glutamate synapses, important for learning, are primarily found on postsynaptic dendritic spines (Sheng and Hoogenraad, 2007) with synapses equipped with both ionotropic and metabotropic glutamate receptors (Hollmann and Heinemann, 1994). The expression trajectories of some ionotropic glutamate receptor subunits, those of AMPA and kainite, do not change during post-natal life. The expression profiles are in fact quite similar to one another, with increasing expression during gestation reaching 100% of maximum around the time of birth and remaining steady thereafter (Kang et al., 2011). Another ionotropic glutamate receptor, N-methyl-D-aspartate receptor (NMDAR), is critically important for the calcium flow in and out of cells that is responsible for both long-term potentiation (LTP) and long-term depression (LTD: Li and Tsien, 2009) while metabotropic glutamate receptors can increase or decrease the excitability of the postsynaptic neuron and regulate postsynaptic protein synthesis through second messenger systems, in effect modulating synaptic plasticity (Hollmann and Heinemann, 1994). Given the interest in NMDARs in schizophrenia, due to the psychotomimetic effect if blocked (Catts and Catts, 2010) and for their role in cognitive ability and synaptic plasticity across development, we will focus on developmental change in NMDARs.

NMDARs are heterotetrameric receptors, comprised of two obligatory NR1 subunits together with two NR2 (A-D) or NR3 (A-B) subunits, with the different assembly of subunits determining the functional differences in receptor properties (Cull-Candy et al., 2001). One of the first studies to examine NR1 mRNA and protein levels across human development in the PFC (Henson et al., 2008) found expression significantly changed across age in a sample from 18 gw to 25-years old. NR1 protein levels were low prenatally, rose to a peak at 11–15 years then reduced slightly in young adults. The mRNA expression followed a similar pattern but with a less marked reduction in adulthood. Two recent transcriptome analyses have also charted NMDAR subunit mRNAs in human prefrontal cortex from early gestation to old age (Colantuoni et al., 2011; Kang et al., 2011; Figure 5). NR1 mRNA expression was generally consistent across post-natal life, confirming our unpublished findings, and not consistent with the putative peak in NMDAR1 early adolescent peak reported earlier (Henson et al., 2008).

In general, in rodents NR2B, 2D, and 3A predominate in early life and 2A, 2C, and 3B are expressed to a greater extent in adults as compared to neonates. Thus, in the developing rodent brain, there is a shift in the ratio of NR2A/NR2B containing receptors during post-natal development in several brain regions, with a higher proportion of NR2A-rich receptors in later life (Ritter et al., 2002; Turman et al., 2002). This ratio switch is also present in the human hippocampus (Law et al., 2003) and the visual cortex (Murphy et al., 2005). NR2B-rich receptors exhibit longer excitatory postsynaptic currents (EPSCs; Monyer et al., 1994; Flint et al., 1997), enabling the coincidence of pre- and postsynaptic events and thus facilitating experience dependent synaptic plasticity in the critical early post-natal period (Philpot et al., 2001). Thus, the decreasing NR2A/NR2B ratio across life may be responsible for the decrease in plasticity seen during maturation and ageing (Crair and Malenka, 1995). In the rat hippocampus, the NR2A/NR2B ratio is believed to mediate the process of synaptic remodeling (Gambrill and Barria, 2011). Higher NR2B expression increases the rate of addition and subtraction of spines, and higher NR2A expression reduces the number of synapses and their volume.

In two recent transcriptome analyses, the prenatal expression of all NMDAR subunit mRNAs were found to be low in both studies, with exception of NR2B mRNA which is high prenatally and peaks shortly before birth (Colantuoni et al., 2011; Kang et al., 2011). While NR2C mRNA expression was steady across most of the life span, it displayed a slight increase after 70 years of age (Colantuoni et al., 2011; Kang et al., 2011). In Henson and colleagues' study of the PFC, NR3A mRNA and protein were very low during prenatal development, at maximum levels in the first year after birth and then declined gradually from ages 1 to 25, to be at 30% of the maximum expression (Henson et al., 2008). We also studied NMDAR mRNA levels in individuals aged from 1 month to 49 years (Choi et al., 2009) and found NR3A (*r* = −0.933) and NR2D (*r* = −0.785) mRNA levels were high within the first few years of human life and were significantly and gradually down-regulated with advancing age. Adult NR3A mRNA expression decreased to 35% of neonatal levels, consistent with the findings of Henson and colleagues (2008). Adult NR2D mRNA decreased to 50% of neonatal levels in our microarray study (Choi et al., 2009). Unlike other NMDAR subunits, NR2D expression is thought to be absent from cell bodies (Thompson et al., 2002) and little is known of how this specific subunit influences the physiology of the receptor, however, there is evidence that recruitment of extrasynaptic NMDARs containing NR2D are important for the expression of LTP in the hippocampus in adult rodents (Harney et al., 2008). Similar to what we find in human cortical development, rodent NR3A expression peaks early in life during the period of synaptogenesis and is down-regulated prior to the critical period of plasticity. This suggests that the critical period for NMDAR mediated plasticity in humans may start around toddlerhood in the prefrontal cortex (Wong et al., 2002; Perez-Otano et al., 2006) at a time where language acquisition and utilization grows at a rapid pace. In rodents, deletion of NR3A increases spine density and leads to failure to maintain juvenile-type synapses (Das et al., 1998; Roberts et al., 2009). Taken together, these results suggest that the major post-natal change in human cortical NMDARs may not be the NR2B to NR2A switch, but rather may feature the down-regulation of NR2D and NR3A more prominently.

The extent to which NMDAR subunit mRNAs are altered in the brains of people with schizophrenia varies with subunit and brain region, however, mRNA changes remain controversial, with decreases (Humphries et al., 1996; Sokolov, 1998; Law and Deakin, 2001; Beneyto and Meador-Woodruff, 2008; Weickert et al., 2012), increases (Akbarian et al., 1996; Dracheva et al., 2001; Schmitt et al., 2010) and no change (Akbarian et al., 1996) observed in NR1. Direct studies of NMDAR protein expression in schizophrenia patients are rare. One study found no significant changes in NR1 or NR2A-D protein levels in schizophrenia patients, however, an alternatively spliced isoform of NR1, NR1^C2^, was increased in the anterior cingulate cortex (Kristiansen et al., 2006). Binding studies also show mixed results depending on brain region and binding site. In the frontal cortex, studies have shown no alteration in binding at the PCP (Dean et al., 1999, 2001; Scarr et al., 2005; Beneyto and Meador-Woodruff, 2008), glycine (Nudmamud and Reynolds, 2001, polyamine or glutamate sites (Beneyto and Meador-Woodruff, 2008) in schizophrenia, while one study found an increase in glycine site binding in the schizophrenia orbital frontal cortex (Simpson et al., 1991) and increases in glycine site binding have also been demonstrated in various other cortical areas including superior temporal cortex (Nudmamud and Reynolds, 2001), somesthetic, visual, and premotor areas (Ishimaru et al., 1992). One study found increased [^3^ H]MK-801 binding in the putamen in schizophrenia (Kornhuber et al., 1989), however, a subsequent study showed no change in binding in the major striatal structures (Noga et al., 1997). We recently demonstrated for the first time a robust decrease in NR1 protein in schizophrenia patients in prefrontal cortex (Weickert et al., 2012). Our study also described decreased expressions of the NR1 and NR2C mRNA subunits in schizophrenia patients (Weickert et al., 2012), which are consistent with an earlier study (Beneyto and Meador-Woodruff, 2008). The Meador–Woodruff group also reported a significant increase in NR3A mRNA (Mueller and Meador-Woodruff, 2004) which could indicate that the NMDARs are in a more immature state and that higher NR3A could interfere with age-appropriate cortical plasticity in schizophrenia. Elimination of interneuronal expression of mature NMDARs post-natally, but prior to onset of adolescence, leads to a schizophrenia-like phenotype in mice (Belforte et al., 2010), suggesting that either the lower levels of the obligatory subunit (NR1) or higher levels of the NR3A subunit could interfere with the maturational changes in NMDARs and contribute to schizophrenia.

### Inhibitory neurotransmitter receptors

The major inhibitory neurotransmitter we will consider here is gamma-aminobutyric acid (GABA) which binds ligand gated ion channel (GABA_A_ receptor) and G-protein coupled receptors (GABA_B_ receptor; Aguayo et al., 2004; Olsen and Sieghart, 2008). GABA is synthesized by two enzymes, GAD_65_ (GAD2) and GAD_67_ (GAD1). GAD_67_ is responsible for 90% of the basal GABA synthesis and is produced at limiting levels in the brain (Asada et al., 1997; Kash et al., 1997). In the human, strong basal expression of GAD_67_ was shown from neonate through adult, with GAD_65_ increasing and peaking in the teenage years (Pinto et al., 2010). However, in the DLPFC, our unpublished microarray data as well as that from Kang et al. (2011) show the mRNA for GAD_65_ peaks around one year of age and stays consistently expressed from infants to adulthood with little or no drop-off. Our microarray data shows a similar pattern for GAD_67_ implying that mRNA for both constitutive and synaptic synthesis of GABA is fairly stable throughout post-natal life. This is interesting as the enzymes responsible for GABA production are expressed at a steady rate despite large changes in distribution, maturational state, and phenotype of the interneuron populations that occur in post-natal human life (see earlier section).

GABA receptors act to hyperpolarize adult neurons, but act to depolarize younger neurons (Flint et al., 1998; Ben-Ari et al., 2007). This reversal is due to the high chloride concentrations found in immature neurons due to high developmental expression of Na-K-Cl co-transporter 1 (NKCC1, also known as SLC12A2: Di Cristo, 2007). NKCC1 is highly expressed during early fetal human development and increases until reaching a plateau in early childhood in the DLPFC (Hyde et al., 2011). The other major potassium-chloride transporter member, KCC2 (SLC12A5), is also expressed in early fetal development, but at much lower levels and also increases before reaching its steady state during early childhood (Hyde et al., 2011). There is uncertainty in establishing an exact crossover point from which GABA switches from excitatory to inhibitory, but it could be as late as 10 years of age (Hyde et al., 2011). The uncertainty can be attributed to two main causes; firstly, the individual variability of the developmental expression of NKCC1 and KCC2, both in Hyde's study and our own unpublished microarray findings, is substantial and thus the point of crossover may occur anywhere from prenatal life to school age when examining the overall profiles. Secondly, it has been shown that individual cells may have differing chloride gradients due to variable subcellular distribution of the two chloride transporters even within the same neuron, particularly at the axon initial segment (Khirug et al., 2008). This would result in GABA having an inhibitory effect on the dendritic portions of a cell but perhaps an excitatory effect at the axon initial segment. In homogenate tissues of the DLPFC, this type of individual cell variation in chloride transporter expression would be diluted but may help to explain NKCC1's continued presence throughout life when GABA action is predominately inhibitory.

Since more comprehensive and validated developmental data are available for the GABA_A_ receptor as compared to the GABA_B_ receptor, we will consider the developmental changes for the GABA_A_ receptor mRNAs here. The GABA_A_ receptor is a pentameric ion channel that is made up from a number of possible subunits, namely 6α, 3β, 3γ, δ, ε, and π subunits (Szabadics et al., 2006; Olsen and Sieghart, 2008). In the DLPFC, the human post-natal developmental change in the GABA_A_ receptor α/β/γ subunits can be divided into two groups (Figure 3). The first comprises the subunits with decreasing mRNA expression over the course of development (α2, α5, β1, γ1, γ3) and the second comprises those with a more dynamic expression pattern (α1, α4, β2, β3, γ2) with an M-shape peaking at the toddler and teenage time periods (Duncan et al., 2010; Fillman et al., 2010). The mature forms (predominant in adulthood) of the receptor subunits such as α1, β2, and γ2 typically have rapid channel opening/closing kinetics and greater sensitivity to GABA leading to greater temporal specificity of inhibitory action (Hevers and Lüddens, 1998; McClellan and Twyman, 1999).

The postsynaptic GABA_A_ receptor α1 and α2 subunits show consistent change in post-natal life with α1 increasing in life with greatest rate of change soon after birth with more gradual increase until young adulthood and with quite dramatic down-regulation of α2 across post-natal life (Hashimoto et al., 2009; Duncan et al., 2010). This leads to an overall increase in the α1/α2 ratio in the prefrontal cortex, and it is interesting that the later peak in this ratio (in teenagers) aligns with the increase in gamma band power found in the prefrontal cortex in humans (Uhlhaas et al., 2009). However, changes in individual receptor subunit mRNAs for the β2, γ2 subunits do not achieve their peak of expression until the teenage/young adult years (Fillman et al., 2010) implying changes in the ratio of other GABA_A_ receptor subunits also may contribute to increasing cognitive capacity of humans through development.

One of the most robust findings in schizophrenia neuropathology is deficits in cortical inhibitory interneurons across several cortical regions (Hashimoto et al., 2008a; Thompson et al., 2009), including DLPFC. Findings include consistent reductions in expression of GAD_67_, which have been recently reviewed (Gonzalez-Burgos et al., 2010). Given the reproducible changes in GAD_67_, one may expect to see correspondingly reproducible changes in the GABA_A_ receptor. While there appears to be a replicable change toward an increase in GABA binding in the cortex of people with schizophrenia (Benes et al., 1992; Dean et al., 1999; Newell et al., 2007; Verdurand et al., 2013), there is less consensus as to changes occurring in the GABA_A_ receptor mRNAs in schizophrenia. In DLPFC from schizophrenia patients, Volk et al. (2002) found an increase in GABA_A_ α2 subunit labeled axon initial segments. However, as the axon initial segment lacks mature KCC2 transporters, the effect of this putative loss of GABA and increase in GABA_A_ α2 is debated (Volk et al., 2002; Szabadics et al., 2006). It is noteworthy that the increased expression of GABA_A_ α2 subunit has been independently replicated (Beneyto et al., 2011), but may not be found in all studies (Duncan et al., 2010). In contrast, decreased expression of the GABA_A_ subunits α1 and α5 in the DLPFC from patients with schizophrenia has been more consistently observed (Hashimoto et al., 2008a; Duncan et al., 2010; Beneyto et al., 2011). The changes occurring in GABA_A_ subunits in schizophrenia (an increase in α2 and a decrease α1) could be interpreted to reflect a cortex that may be held in a state of immaturity into adulthood. This concept is interesting as it fits with a number of other changes that would also indicate that the schizophrenia cortex could resemble a more immature cortical state, like increased NR3A, decreased parvalbumin and more calbindin with less cholecystokinin expression as just a few other notable examples consistent with this model.

### Dopamine receptors

**Figure 6:**
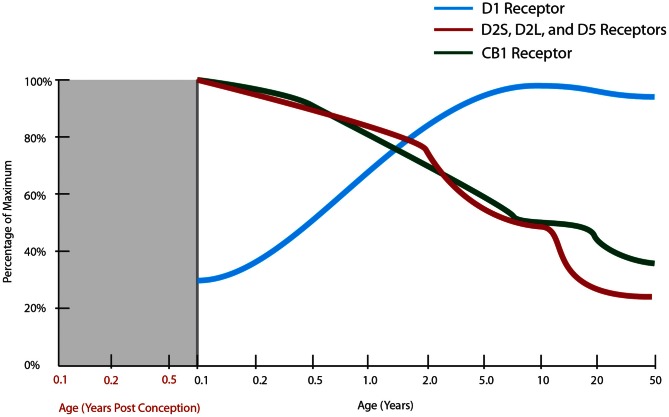
**Normal developmental trajectories of dopamine and cannabinoid expressions in the human prefrontal cortex.** Expression of dopamine receptor, DRD1 suggests an inverted U-shape with increasing expression of receptors until the mid-twenties after which there is a slow decline (Rothmond et al., 2012). The DRD2 isoforms and DRD5 show an inverse pattern, peaking prenatally with a continuous decline in expression throughout life, excepting a small increase in the early twenties (Rothmond et al., 2012). The expression of DRD4 as determined by *in situ* hybridization is stable across the lifespan (Weickert et al., 2007). Measurement of this receptor by quantitative PCR has been quite variable and no statistical differences in expression across age groups have been detected (Rothmond et al., 2012). Cannabinoid receptor mRNA has its highest measured expression at the time of birth and shows a stepwise decline with age, reaching 30% of maximal expression in middle age (Long et al., 2012).

Functions of the DLPFC, like working memory, cognitive control, and reasoning (Goldman-Rakic, 1996; Nieoullon, 2002; Glickstein et al., 2005; Mizoguchi et al., 2009) are impacted in schizophrenia and are sensitive to changes in cortical dopamine (Williams and Goldman-Rakic, 1995; Lewis, 1997; Romanides et al., 1999) As these cognitive functions change across development, it is of interest to consider how dopamine receptors may change as humans grow and mature. Cortical dopamine receptors fall into two distinct categories. DRD2-like receptors [DRD2 (S = short and L = long), DRD4] and DRD1-like receptors (DRD1, DRD5: Monsma et al., 1989; Missale et al., 1998). The expression of DRD2 (both forms), DRD4, DRD5 mRNAs all display a predominate pattern of developmental change with highest levels of expression in infants that then declines with age (Weickert et al., 2007; Rothmond et al., 2012; Figure 6). Interestingly, the opposite pattern of expression for both mRNA and protein is observed for DRD1 making DRD1 unique among the cortical dopamine receptors in terms of its developmental expression profile and suggests that there is an increased role of DRD1 as the human cortex matures (Weickert et al., 2007; Rothmond et al., 2012; Figure 6). By early adulthood DRD1 appears to be the most prevalent receptor in the human PFC (Lidow and Rakic, 1992; Meador-Woodruff et al., 1997) and DRD1 is critical to PFC cognitive functioning and in particular working memory (Arnsten et al., 1994). Thus, the gradual increase in DRD1 protein levels from pre-adolescence into young adulthood happens during a time in development when the cortex functionally matures. It coincides with improved performance on a cognitive test thought to tap into a core cognitive deficit in schizophrenia, the Wisconsin Card Sorting Test, which measures behavioral flexibility and working memory (see earlier section), suggesting that DRD1 function may have a particularly salient role in working memory. Indeed, DRD1 receptors in adolescent rodents were shown to interact with NMDARs on pyramidal neurons to increase the likelihood that they would be driven into “up-states” thought to be necessary for working memory function (Tseng et al., 2007).

Electrophysiological studies show that in cortical pyramidal neurons, DRD1-like receptor agonism is excitatory (enhancing NMDA effects) and DRD2-like receptor agonism is inhibitory (attenuating AMPA and NMDA response: Wang and O'Donnell, 2001; Tseng and O'Donnell, 2004). In interneurons, DRD1-like receptors are excitatory throughout life (Tseng and O'Donnell, 2007), whereas DRD2-like receptors are weakly inhibitory in juvenile rodents but switch to being strongly excitatory in adult rodents, i.e., strongly increasing inhibitory firing of interneurons onto pyramidal neurons (Tseng and O'Donnell, 2004, 2007). In conditions of high dopamine, this leads to a greater contrast between pyramidal neurons firing due to strong excitatory input and the suppression of background activity of surrounding pyramidal cells (see O'Donnell, 2010 for a review). The cellular mechanisms underlying the switch from inhibitory to excitatory effect of DRD2-like receptors in interneurons are unclear (O'Donnell, 2010), but it yet again implicates peri-adolescent changes in cortical inhibitory circuits as critical foci of research for those seeking to understand the neurodevelopmental neuropathology of schizophrenia.

It is interesting to note that the other dopamine receptor linked to neuronal excitation, DRD5, has a developmental expression pattern opposite to that of DRD1 expression with the highest levels of expression within the first year of life, given that both receptors are nearly indistinguishable pharmacologically. DRD5 is consistently co-localized with DRD1 in the PFC (Bordelon-Glausier et al., 2008) and both are found on pyramidal neurons that are the neural substrates of working memory (Goldman-Rakic et al., 1989). Dopamine has a 10-fold higher affinity to DRD5 compared to DRD1 (Sunahara et al., 1991) suggesting DRD5 even when expressed at lower levels than DRD1 may be functionally important to cognitive processing of working memory (Amico et al., 2007). These results suggest that DRD5 may be the dominant receptor working in opposition to DRD2 early in life in infancy and toddlerhood, and that there may be a developmental switch that occurs such that DRD1 plays a more salient role in dopamine mediated excitation in neural processing during childhood and adolescence.

Schizophrenia studies investigating mRNA expression of all five dopamine receptors have found no change in DRD3, DRD4, and DRD5 in the DLPFC (Meador-Woodruff et al., 1997; Zhan et al., 2011). However, given the timing of late developmental up-regulation and the putative working model of an immature cortex found in schizophrenia, one may predict that DRD1 should be decreased in schizophrenia. While several *in vivo* binding studies support this (Okubo et al., 1997; Kosaka et al., 2010), only one post-mortem study has found a decrease in cortical DRD1 binding (Hess et al., 1987), which appears to be reflected in DRD1 mRNA expression levels (Meador-Woodruff et al., 1997; Zhan et al., 2011). There is, however, more support for the idea that DRD2 can be elevated in schizophrenia DLPFC (Hess et al., 1987; Kestler et al., 2001; Tallerico et al., 2001). Both DRD1 and DRD2 continue to be expressed throughout life and the interplay of the DRD1 and DRD2 receptors are integral to excitation and inhibition of adult cortical neurons (Onn et al., 2005; So et al., 2005; Cropley et al., 2006; Floresco and Magyar, 2006; Winter et al., 2009). Identifying the cellular localization of this up-regulation of DRD2 would be important for determining whether the increased DRD2 is perhaps counteracting DRD1 effects on pyramidal neurons or bolstering the inhibitory drive of interneurons in schizophrenia prefrontal cortex.

## Psychopharmacological responses to drugs of abuse

When considering schizophrenia from a developmental standpoint, it is of interest to consider that the time of onset of schizophrenia is also when humans are, for the first time, more likely to come in contact with mind-altering drugs. The adolescent brain seems to have unique responses to psychoactive drugs compared with children and adults. Adolescent mammals are less sensitive to the locomotor enhancing and cortisol-releasing effects of stimulants such as amphetamine and cocaine compared with adults (Laviola et al., 2002; Zombeck et al., 2010). Furthermore, the behavioral sensitization to stimulant drugs observed in adults can be absent in adolescent rodents (Laviola et al., 1995; Adriani et al., 1998). In contrast, effects of dopamine D2 receptor antagonism such as catalepsy, or prolonged fixed body posture, are more prominent in adolescents than adults (Spear et al., 1980; Campbell et al., 1988) perhaps due to the late up-regulation of D1 receptor and the changing D2R/D1R ratio, although these behaviors are thought to be more related to subcortical dopamine action than cortical dopamine action. Also, the ability of dopamine auto-receptors to suppress subcortical dopamine release matures during adolescence (Hedner and Lundborg, 1985), which might alter dopamine release following stimulant administration. Many people with schizophrenia show increased susceptibility to behavioral and dopamine-releasing effects of amphetamine (Lieberman et al., 1987; Laruelle et al., 1996), and hypersensitivity to typical psychostimulant effects such as locomotor hyperactivity is a benchmark of face validity of animal models of schizophrenia (Powell and Miyakawa, 2006).

Non-competitive NMDAR antagonists such as ketamine and phencyclidine produce psychotic and cognitive symptoms in healthy volunteers, exacerbate schizophrenia symptoms in patients and exert greater cognitive impairment in patients with schizophrenia as compared to healthy controls (Yago et al., 1981; Lahti et al., 1995, 2001; Malhotra et al., 1997). Ketamine and phencyclidine do not readily produce hallucinations in children, despite doing so in normal adults (Hirsch et al., 1997) perhaps due to post-natal difference in NMDAR subunit composition in children compared to adults (see earlier). Perinatal NMDAR antagonist administration induces impairments in attention, working memory, executive function, and social cognition in adulthood (Du Bois and Huang, 2007). In addition, these changes may be more pronounced in rodent pups treated perinatally than in animals exposed to NMDAR antagonists at later developmental stages (Mouri et al., 2007). This suggests that age of exposure to drugs may interact with normal developmental changes in NMDARs to bring about differential cortical effects and can result in distinct behavioral effects.

Compared with adults, adolescent rats find repeated administration of the active ingredient in cannabis, delta-9-tetrahydrocannabinol (THC), less aversive but are more likely to show residual deficits in social interaction and working memory after single or chronic THC exposure (Quinn et al., 2008). Similarly, adolescent rats show higher tolerance to the effects of alcohol (Swartzwelder et al., 1998) and are more likely to “binge-drink” than adults (Hargreaves et al., 2009a), but may also be more vulnerable to neurobiological change following alcohol exposure (Hargreaves et al., 2009b). Thus, while some drugs are more rewarding in younger individuals, this can lead to an increased potential for drug dependence (Quinn et al., 2008; Hargreaves et al., 2009a) and the likelihood of other detrimental effects of drugs of abuse. In humans, cannabinoid CB_1_ receptor mRNA is highly expressed in the DLPFC from birth until toddlerhood, when it begins to fall rapidly until adolescence and is reduced to a trough by adulthood (Long et al., 2012; Figure 6). Synthetic capacity for the major cortical endogenous cannabinoid neurotransmitter peaks at adolescence, but is followed by a reduction in adulthood (Long et al., 2012). This identifies significant turning points in the prominence of normative endogenous cannabinoid signaling and the subsequent developmental regulation of inhibitory neurotransmission, since the majority of CB_1_ receptors are localized to GABAergic terminals (Eggan et al., 2010a). Thus, the increased susceptibility to psychotomimetic effects of THC in people with schizophrenia (D'Souza et al., 2005) may be due to aberrant development of the endocannabinoid system during a critical period of change, and indeed numerous reports show that CB_1_ receptors are altered in schizophrenia (Zavitsanou et al., 2004; Newell et al., 2006; Eggan et al., 2008, 2010b; Dalton et al., 2011). While both increases and decreases in CB_1_ receptors are reported depending on brain region and if receptor binding, mRNA or protein is measured, it is possible that subtypes of schizophrenia may present with different alterations in the endogenous cannabinoid system (Dalton et al., 2011). In general, observations of the changes in the response to psychoactive drugs during development can be used together with developmental processes to understand not only detrimental effects of these drugs, such as psychotic reactions, but also the way in which development may have gone awry in schizophrenia.

## Conclusion

The schizophrenia prodrome can occur during the adolescent years when the human prefrontal cortex is undergoing molecular and functional change (Cornblatt et al., 2003). Deficits in higher order cognitive abilities remain among the most important causes of persistent functional disability in schizophrenia. Therefore, closer examination of the normal developmental trajectories of these cognitive functions and their underlying neural substrates should be considered in the context of schizophrenia in order to adequately identify the key risk factors and determinants of abnormal cortical development in schizophrenia. To date, the predominant neurodevelopmental model used to underpin the emergence of schizophrenia during adolescence is one that posits synaptic regression as the dominant feature of adolescence, a theory based largely on work done over 25 years ago. We suggest that this view is an overly simplistic and a temporally inaccurate way to frame adolescent development of the human cortex. When considering schizophrenia in the context of neurodevelopment and giving appropriate emphasis on the more recently described post-natal developmental changes, a more modern, dynamic, and complex picture emerges. While many developmental changes are yet to be confirmed, many others have been replicated and these provide ample scope for changes in risk genes and environments to alter post-natal developmental trajectories. We also suggest that while some processes may be attenuated with human post-natal brain growth, many processes are accelerated and others are more accurately viewed as in an active growth phase and more dynamic state of change. Thus, it may be inaccurate to consider the major developmental event of the human cortex to be one of synaptic regression. Certainly, there is an overwhelming increase in brain size, in interneuron differentiation, in synaptic molecule expression, and in GABA and dopamine receptor mRNA levels that would indicate that gains in synaptic strength predominate in early life and continue during childhood. What appears to be the major developmental switch at adolescence is the slowing of the exuberant growth period typical of infants, toddlers, and children. We suggest that, when theorizing about the neurodevelopmental basis of schizophrenia, schizophrenia could be considered to result from a failure to reach the final state of cortical maturation resulting in retainment of an immature cortex (at least transiently) rather than resulting from excess of adolescent synaptic pruning. Further, it is clear that adolescence is a time of dynamic brain change and that schizophrenia could be viewed as resulting from a destabilization of a normal adolescence process. Since the adolescent brain is in a state of flux it may be possible to stabilize the adolescent brain of those at risk so that any disruption is only transitory and so that chronic schizophrenia does not emerge (McGorry, 2011).

### Conflict of interest statement

The authors declare that the research was conducted in the absence of any commercial or financial relationships that could be construed as a potential conflict of interest.
